# Durability Properties of Lightweight Foamed Concrete Reinforced with Lignocellulosic Fibers

**DOI:** 10.3390/ma15124259

**Published:** 2022-06-16

**Authors:** Md Azree Othuman Mydin, Mohd Nasrun Mohd Nawi, Ruba A. Odeh, Anas A. Salameh

**Affiliations:** 1 School of Housing, Building and Planning, Universiti Sains Malaysia, Gelugor 11800, Penang, Malaysia; 2 Disaster Management Institute (DMI), School of Technology Management and Logistics, Universiti Utara Malaysia, Sintok 06010, Kedah, Malaysia; nasrun@uum.edu.my; 3 Department of Allied Engineering Sciences, Faculty of Engineering, The Hashemite University, Zarqa 13133, Jordan; rubaa@hu.edu.jo; 4 Department of Management Information Systems, College of Business Administration, Prince Sattam Bin Abdulaziz University, Al-Kharj 11942, Saudi Arabia; a.salameh@psau.edu.sa

**Keywords:** lightweight foamed concrete, water absorption, lignocellulosic plant fibers, jute, ramie, durability properties

## Abstract

Worldwide concern and ascendancy of emissions and carbon footprints have propelled a substantial number of explorations into green concrete technology. Furthermore, construction material costs have increased along with their gradual impact on the environment, which has led researchers to recognize the importance of natural fibers in improving the durability and mechanical properties of concrete. Natural fibers are abundantly available making them relatively relevant as a reinforcing material in concrete. Presently, it should be recognized that most construction products are manufactured using resources that demand a high quantity of energy and are not sustainable, which may lead to a global crisis. Consequently, the use of plant fibers in lightweight foamed concrete (LFC) is deemed a practical possibility for making concrete a sustainable material that responds to this dilemma. The main objective of this study is to investigate the effect of the addition of lignocellulosic fibers on the performance of LFC. In this investigation, four different types of lignocellulosic plant fibers were considered which were kenaf, ramie, hemp and jute fibers. A total of ten mixes were made and tested in this study. LFC samples with a density of 700 kg/m^3^ and 1400 kg/m^3^ were fabricated. The weight fraction for the lignocellulosic plant fibers was kept at 0.45%. The durability parameters assessed were flowability, water absorption capability, porosity and ultrasonic pulse velocity (UPV). The results revealed that the presence of cellulosic plant fibers in LFC plays an important role in enhancing all the durability parameters considered in this study. For workability, the addition of ramie fiber led to the lowest slump while the inclusion of kenaf fiber provided optimum UPV. For porosity and water absorption, the addition of jute fiber led to the best results.

## 1. Introduction

The utilization of lightweight foamed concrete (LFC) in building construction and housing development has expanded considerably throughout the world [1]. LFC is a cement paste, recognized as lightweight concrete, in which air voids of approximately 0.1 mm to 1.0 mm in diameter are evenly scattered in the cement paste by a suitable foaming agent [2]. LFC has great flowability, is low in density, and provides good sound absorption and exceptional thermal performance [3]. With proper control of the quantity of foam added to the mortar slurry, an extensive variety of densities can be produced ranging between 400 and 1900 kg/m^3^ [4]. LFC is regarded as an economical substitute in the production process pertaining to large-scale lightweight construction components, such as road embankment infills, wall panels, structural elements, and filling grades due to its easy and simple manufacturing process, which encompasses all stages, right from the start of the manufacturing plants to the final position [5,6,7].

Most of the existing research pertaining to concrete concentrates on high-performance concrete, and focuses on the demanding performance needs, including the durability, mechanical and thermal performances [8]. There are not many research studies focusing on LFC’s durability characteristics, which include relatively inadequate ductility, low tensile strength, brittleness as well as low resistance toward cracking. LFC also possesses internally induced cracks, and the propagation of microcracks leads to brittle fractions resulting in poor tensile strength. With regard to LFC, the development of structural cracks occurs even before loading because of the high drying shrinkage and other various reasons. After applying the load, the internal cracks tend to propagate and grow because of stress, resulting in the creation of further cracks [9]. These cracks as they progress cause inelastic deformation in LFC. When the loading is less than the critical dynamic stress, the hysteretic curve is flat and thin, specifying that the viscosity of LFC is low and indicating that its damping ratio and energy dissipation are insignificant. The LFC primarily creates elastic deformation under the loading force, and the reciprocating deformation change in an elastic state uses almost no energy. When the loading strength is larger than the critical dynamic stress, the form of the hysteretic curve tends to be thicker [10]. Under cyclic loading, besides the elastic deformation energy consumption, the LFC specimen generates cumulative plastic deformation, and the energy loss triggered by solid particle friction grows steadily. Hence, the hysteretic curve grows wider and has a tendency to be as smooth as the hysteretic curve of the viscoelastic body. When LFC is utilized as the filling material, the actual engineering condition does not allow it to consume energy caused by the damage of the material itself [11].

Introducing evenly distributed and small, tightly-spaced natural lignocellulosic fibers in LFC helps prevent the development of cracks and enhances the concrete’s static and dynamic characteristics [12,13]. This is also termed fiber-strengthened LFC and can be defined as LFC that consists of fibrous materials to improve its structural performance [14]. This includes evenly distributed, short discrete fibers that are oriented randomly [15]. The most commonly used fibers for concrete strengthening are glass, carbon and steel which promote high strength and modulus for structural functions [16]. However, these fibers have a reasonably high cost compared to natural fibers. These conditions have stemmed from an increase in experimental investigations into the utilization of natural fibers as reinforcement in fiber-reinforced concrete [17]. Lignocellulose plant fibers seem to be the most favorable material because they are economical and amply available in most developing countries [18].

Natural lignocellulosic plant fibers are of the same order of importance as aggregate additions. The inclusion of these fibers can improve strength and diminish plastic and drying shrinkages by stopping the spread of cracks [19]. The utilization of steel strengthening concrete has overcome the drawbacks of insufficient tensile strength [20]. Although this doesn’t entirely solve the problem of cracks due to drying and plastic shrinkage during weathering conditions [21]. This led to the investigation of numerous methods to adopt natural short fibers as reinforcement in cement-based materials [22]. The inclusion of steel fiber decreased the formation of cracks, however, over a long period, steel becomes rotted due to several situations [23]. This made the need for insight into the utilization of numerous organic and inorganic fibers which are economical and eco-friendly [24]. Compared to natural fibers, plant-based natural organic fibers are very renewable and eco-friendly and have low production costs [25,26]. The primary markets pertaining to the existing natural plant fibers are mostly based in developing countries that have local availability and include low acquisition costs. However, in developed countries, the employment of natural fibers is still limited since they are yet to be fully accepted as a substitute for synthetic fibers [27]. From the above review, there is a huge potential for utilizing natural lignocellulosic plant fibers in cement-based materials such as LFC. Hence, this research project was aimed at identifying the potential uses of natural lignocellulosic plant fibers to fortify LFC as well as its durability characteristics. Four types of natural lignocellulosic plant fibers were considered as an additive in LFC: kenaf fiber, ramie fiber, jute fiber and hemp fiber.

## 2. Materials and Methods

**Figure 1:**
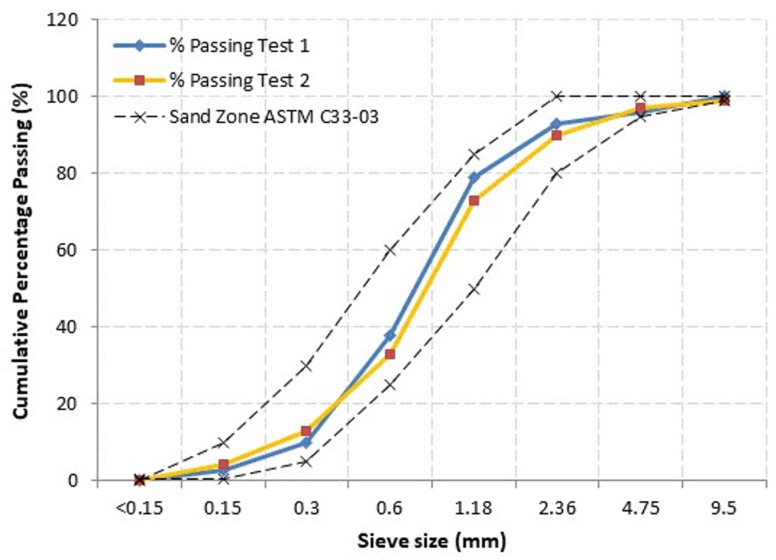
Fine sand grading curve.

**Figure 2:**
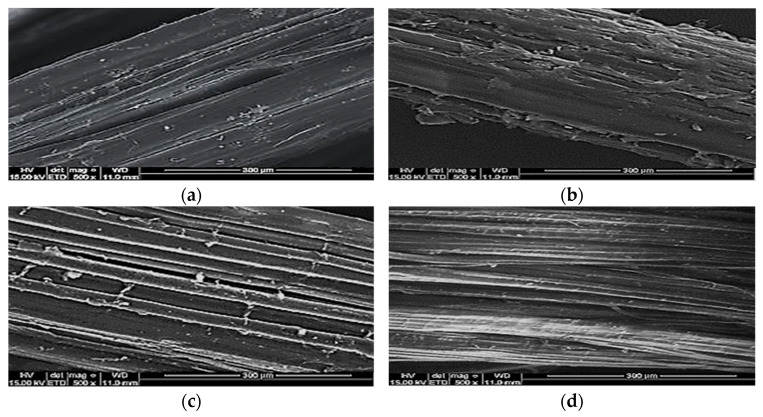
SEM micrograph of lignocellulosic plant fibers. (**a**) kenaf fiber; (**b**) hemp fiber; (**c**) ramie fiber; (**d**) jute fiber.

In this research study, the authors employed Ordinary Portland Cement (OPC) compliant with BS12 [28]. Decent condition cement was used in this study and was kept in a covered area. Next, naturally fine sand was employed as fine aggregate. The fine sand had a 600-micron sieve, a width limit of 2 mm and a passage of 60–90%. Figure 1 shows the fine sand grading curve employed in this investigation. The sand’s suitability complied with BS822 [29]. Table 1 and Table 2 show the physical property of OPC and fine sand; and the chemical composition of cement used in this study, correspondingly. A protein surfactant, named Noraite PA-1, was employed. This Noraite PA-1 was selected as the foaming agent because of its good stability as well as smaller bubble size, which results in the formation of a stronger bubble bonding structure versus a synthetic surfactant. Next, clean and clear tap water was employed for preparing the mortar, the curing work and mixing the LFC. For this research study, a 0.45 water-cement ratio was employed as this ratio can realize reasonable workability, with regards to previous research. Finally, the employed natural lignocellulosic fibers were ramie, jute, hemp and kenaf, which were freshly procured from an industrial unit post-processing. These fibers were cleaned until they were free of grease and then placed under the sun to dry. Table 1, Table 2 and Table 3 show the chemical composition, physical properties and mechanical properties of natural lignocellulosic fibers utilized in this study. To characterize the structure of natural lignocellulosic fibers’ surface, a secondary electron mode was employed for electron microscopy. A vacuum was used to dry the fiber specimens (ramie, kenaf, jute and hemp), which were then coated with a thin gold layer before observation to remove the impacts of charging during the collection of images. Figure 2 shows the SEM images of different types of lignocellulosic plant fibers employed in this study. Table 4 and Table 5 demonstrate the physical and mechanical properties of natural lignocellulosic fibers respectively.

## 3. Mix Design and Sample Preparation

A total of ten LFC mixes of 700 and 1400 kg/m^3^ densities were fabricated. The weight fraction of the fibers applied in this research was 0.45%. In this research study, a weight fraction of 0.45% was used as, during the pilot study, the researcher identified that introducing fiber with greater than 0.45% weight fraction resulted in agglomeration as well as non-uniform dispersion of fibers when carrying out mixing. Moreover, when adding fiber of a 0.45% weight fraction to LFC, the best improvement was achieved with regard to durability characteristics. A sand-cement ratio of 1:1.5 was employed while a 0.45 water-cement ratio was kept constant for all the mixtures. The mix proportions pertaining to this study have been listed in Table 6.

The LFC mixing method utilized in this research used the pre-foamed approach. This method was in accordance with ASTM C796/C796M-12 [30] using a protein-based surfactant. The surfactant was diluted in clean water at a ratio of 1:30 (surfactant: water). The diluted surfactant was stirred through a foam generator by an air compressor with an air pressure of about 480 kPa to create a stable foam in the range of 65–85 g/L. Next, the base mix material comprising of cement, sand and water was slowly mixed in a tilting concrete mixer. The lignocellulosic fibers were gradually introduced into the homogeneous mix. The consistency of the base mix was determined through visual inspection and the use of the Brewer open-ended cylinder spread test. The required spreadability before the introduction of the foam was between 200–250 mm. After reaching the required consistency, a one-liter cup was used to measure the wet density of the base mix. This was used in the calculation of the volume of foam required. The required volume of foam was produced and introduced into the base mix. It was allowed to mix for 3 ± 2 min. The LFC fresh mix was then measured using a 1-liter cup for the actual wet density before being placed into the molds. The desired wet or plastic density was achieved if the difference in density between the wet or plastic density and the dry target density was between 100 ± 50 kg/m^3^. The LFC fresh mix was cast on the prepared oiled molds and allowed to set for 24 h before demolding. Demolding was conducted, and specimens were moist-cured using plastic wrapping. Before testing, the specimens were unwrapped and put in an oven to be oven-dried for 24 h at 100 ± 5 °C.

## 4. Test Methods

### 4.1. Slump Test

**Figure 3:**
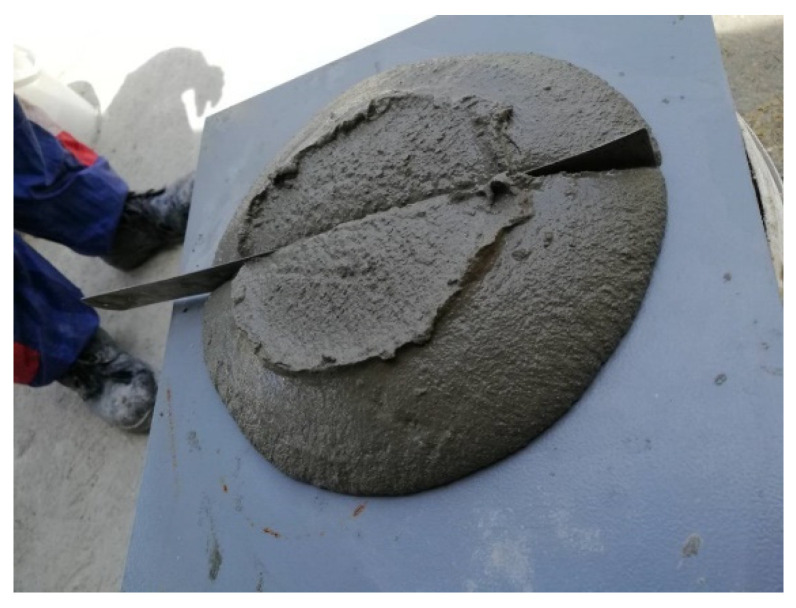
Slump test of fresh LFC.

As a fresh concrete characteristic, the slump test (flowability) of LFC was evaluated. The slump represents the workability pertaining to the mix that corresponds to ASTM C 1611 as shown in Figure 3. To place the fresh LFC mix into the mold, a filling cone was employed. Without tamping or vibration, the LFC was mounted in one lift. The mold was lifted to evenly distribute the concrete. When the spreading stopped, measurement of the two diameters pertaining to the concrete mass was performed in approximate orthogonal directions; the slump flow can be defined as the average of the two diameters.

### 4.2. Ultrasonic Pulse Velocity (UPV) Test

**Figure 4:**
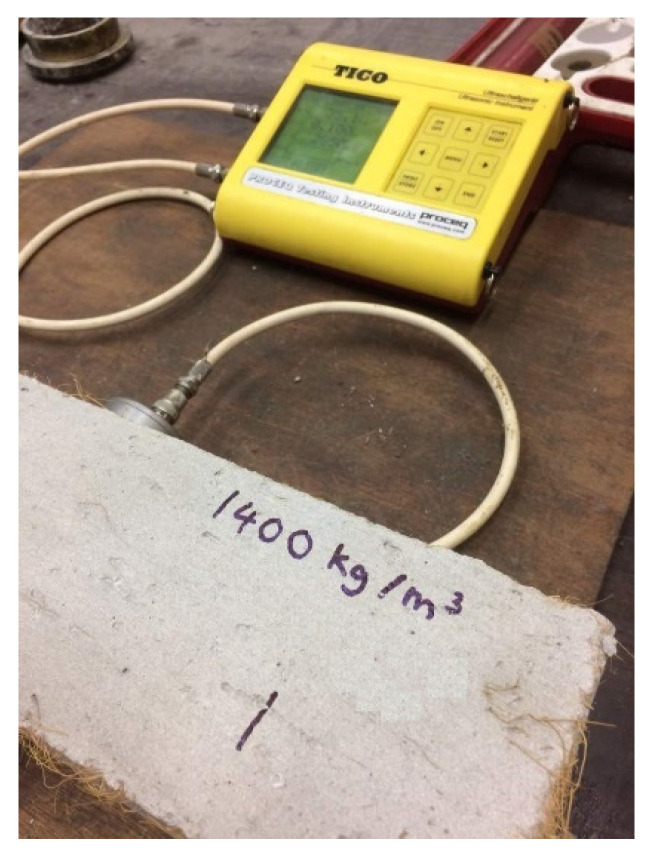
Ultrasonic pulse velocity test of LFC specimen.

Evaluation of the UPV test was done via measurement of the propagation velocity pertaining to a transmitted longitudinal ultrasonic pulse in the cross-sectional area. Measurement of the mortar prisms was done with dimensions of 100 mm × 100 mm × 500 mm. To evaluate the transmission of the ultrasonic pulse, an electro-acoustical transducer was used as shown in Figure 4. It is held in contact with the help of an electrical signal via a second transducer post traveling of the pulse through a known path length in the specimen. The transmitted time and velocity were demonstrated by the electromagnet transducer. The standard procedures were carried out based on the standard laid out in BS EN 12504-4 [31]. Construction of a mortar prism with the dimensions of 100 mm × 100 mm × 500 mm was accomplished for all mixed designs and assessed on day 28.

### 4.3. Porosity Test

**Figure 5:**
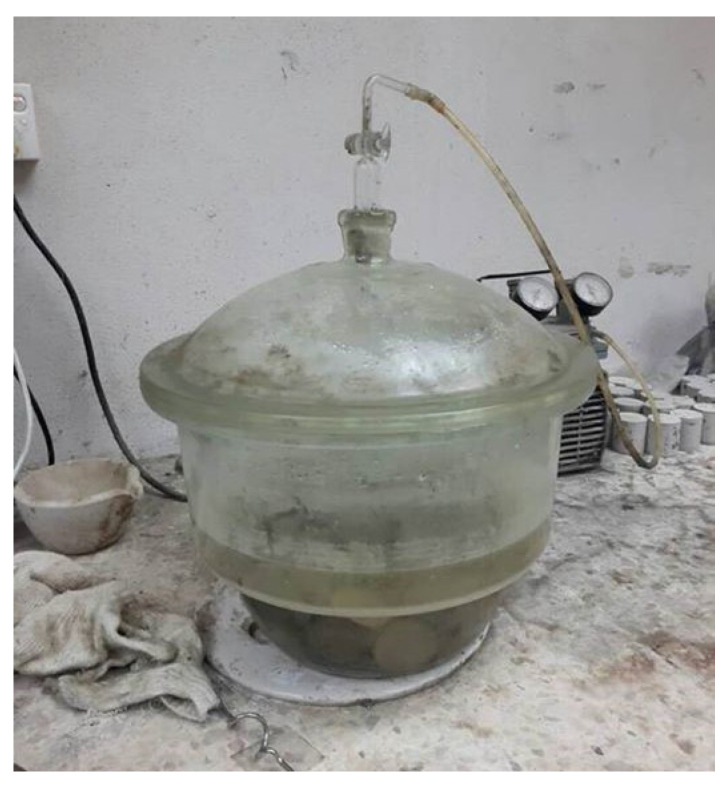
Porosity test via vacuum saturation method.

The vacuum saturation method was employed to execute the porosity test as displayed in Figure 5. On day-28, this test was carried out by introducing the LFC specimens to a vacuum desiccator. This test aims to establish the percentage of air voids with regard to the LFC specimens, which can directly impact the durability performance. From each batch, three LFC specimens possessed a height of 50 mm and diameter of 45 mm, which were kept inside an oven to eliminate moisture for 72 h or until there were no changes recorded in terms of the weight. Then, cooling of each specimen was performed and its weight was recorded as *W_dry_*. In a vacuum chamber, the specimens were kept fully immersed until the 72nd hour or until there no bubbles could be seen. Recording of the weights of the specimens in the air (*W_s,w_*) as well as in the water (*W_s,a_*) was performed. Equation (1) was employed in order to measure the porosity percentage with regard to the LFC.

(1) $$\;\;\;Total\;porosity,\;\left(\%\right)=\left(\frac{W_{s,a}-W_{dry}}{W_{s,a}-W_{s,w}}\right)\times 100\%$$

where,

*W_s,a_* = weight of saturated sample in air
*W_dry_* = weight of oven-dried sample
*W_s,w_* = weight of saturated sample in water.

### 4.4. Water Absorption Test

**Figure 6:**
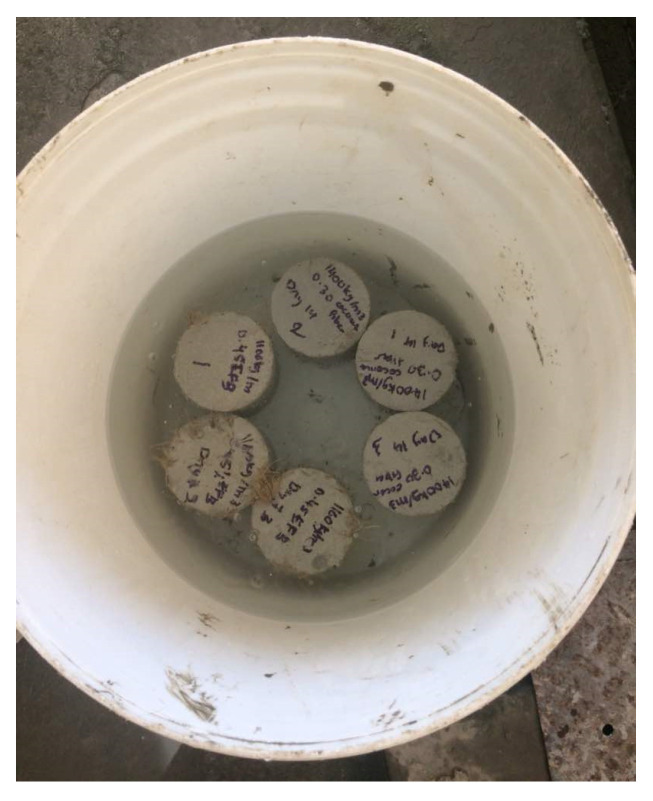
Water absorption test.

A water absorption test is regarded as important for measuring the absorbed water percentage by the LFC specimens at a specific time as demonstrated in Figure 6. Additionally, a higher water percentage absorption in LFC could also impact its porosity and density. As stated in BS 1881-122, the water absorption test was executed [32]. Cylindrical-shaped samples of 75 mm in diameter and 100 mm in height were prepared. When the aging day of the test arrived, from each batch, three specimens were unwrapped and placed under the oven for 72 h to dry. In the next step, the weight of each cooled, oven-dried specimen was recorded as *W_d_*, and then the specimens were completely immersed in a water tank for almost 30 min. Later, any excess water present on the test specimen was removed via a dry cloth and its weight was recorded as *W_s_* in a saturated condition. The water absorption has been signified as a percentage as *W_a_*, which was calculated by employing Equation (2). As the final result, the average water absorption pertaining to these three samples was considered.

(2) $$Water\;absorption,\;\left(\%\right)=\left(\frac{W_{s}-W_{d}}{W_{d}}\right)\times 100\%$$

where,

*W_s_* = Saturated surface dry weight
*W_d_* = Oven-dried weight.

## 5. Results and Discussion

### 5.1. Workability 

**Figure 7:**
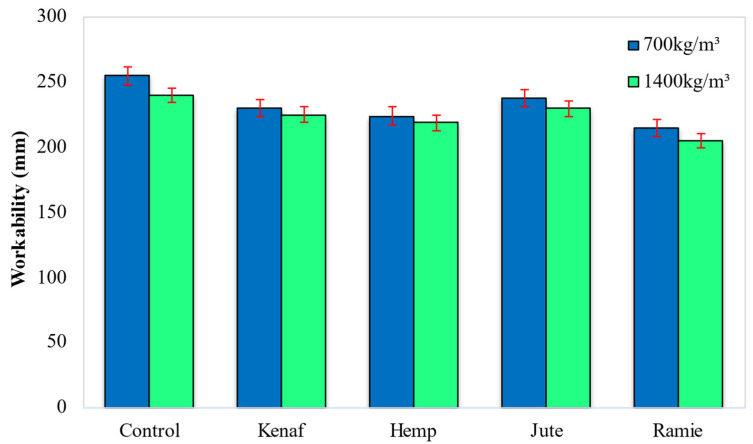
Influence of different types of fiber on the workability of fresh LFC mixes.

During the preparation stage of LFC, the slump of each mixture was measured; these results are summarized in Figure 7. The control mix (no fiber) demonstrated a slump of 255 mm and 240 mm for 700 and 1400 kg/m^3^ densities, respectively. Assessing the control LFC slump with the other mixes, the inclusion of all types of natural fibers in LFC showed a reduction in the workability of the LFC mixtures. The decrease in LFC workability was probably instigated by the intensification of friction between the components of the mixture and the reduction of the free water content as these natural fibers tend to absorb water due to their hydrophobic nature [33]. This explains the increase in the slump corresponding to the inclusion of natural fibers, as their surface presents significant porosity which, at the same time, affects the bond to a hydrophobic matrix and reduces the mechanical properties of the resulting composite material [34,35]. In addition, the lignocellulosic fiber’s large specific surface absorbs more of the cementitious matrix around the fibers and, subsequently, enhances the viscosity of LFC, which causes a slight decrease in the values of the spreadability. These natural fibers are passive materials that do not chemically interact with the foaming agent (surfactant) and water in the foam system. These natural fibers will push apart the foam films with the creation of distinctive networks in their thickness. This will offer a rise in the workability of the LFC mixes due to the free movement of the scattered phase over the volume of the aqueous dispersal medium, including amongst the bubbles. Among the four fibers considered in this study, the addition of jute fiber led to optimal workability (slump) of 238 mm and 230 mm for 700 kg/m^3^ and 1400 kg/m^3^ densities, respectively, followed by kenaf fiber (230 mm and 225 mm), hemp fiber (224 mm and 219 mm) and ramie fiber (215 mm and 205 mm). These slump results can be associated with the cellulose content of the fibers as shown in Table 3. Fiber with a high amount of cellulose tends to absorb more free water in the mix and demonstrate a low slump. With reference to Table 1, ramie fiber has the highest cellulose content of 70.1%, followed by hemp fiber (65.1%), kenaf fiber (63.5%) and jute fiber (47.8%).

### 5.2. Ultrasonic Pulse Velocity

In order to establish the quality of LFC, an ultrasonic pulse velocity test was executed. The UPV enables the assessment of the material homogeneity and facilitates the identification of defects. The UPV technique is employed when the material propagates via a high-frequency sound wave. Wave speed changes as per the material density, which enables the estimation of porosity as well as the detection of discontinuity. The quality of the majority of building materials is based on their rigidity. To assess this, the UPV can be employed to assess the quality of concrete structures, compressive strength and modulus of elasticity as well as to estimate the mechanical characteristics. Figure 3 displays the results pertaining to the ultrasonic pulse velocity of LFC when different kinds of natural lignocellulosic fiber are introduced.

**Figure 8:**
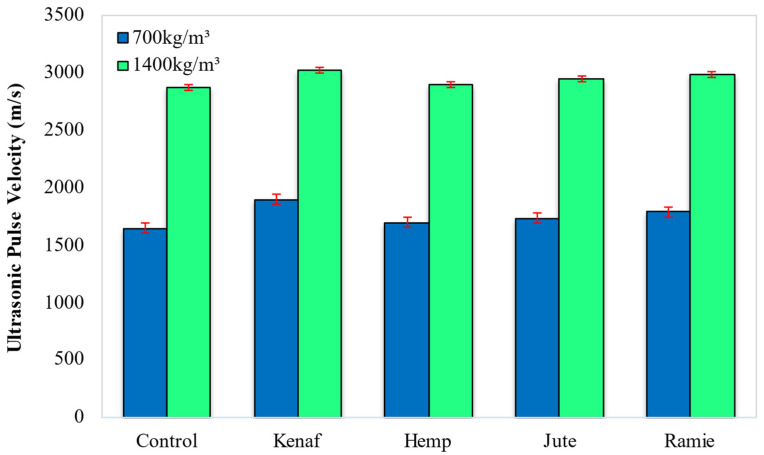
Effect of different types of fiber on ultrasonic pulse velocity of LFC.

According to Figure 8, the optimal result of the UPV was attained with the inclusion of kenaf fiber. The UPV result with the inclusion of kenaf fiber was the highest (1897 m/s and 3025 m/s for 700 kg/m^3^ and 1400 kg/m^3^ densities, respectively), followed by ramie fiber (1788 m/s and 2988 m/s), jute fiber (1734 m/s and 2945 m/s) and lastly the hemp fiber (1695 m/s and 2902 m/s). The control specimen only attained ultrasonic pulse velocity readings of 1644 m/s and 2876 m/s for 700 kg/m^3^ and 1400 kg/m^3^ densities, respectively. This illustrates that the control specimen was deemed a poor quality of LFC compared to LFC with the addition of natural lignocellulosic fibers. Thus, the inclusion of fiber can improve the quality of LFC. The UPV results that were obtained in this study can be associated with the fibers’ cross-section diameters. The fibers’ cross-section diameter in a hardened cement matrix can also impact the LFC’s UPV. In a fiber’s cross-section of larger diameter, individual cells can fill the spaces between the void cells pertaining to LFC and result in a denser composite, which can simultaneously enhance the LFC’s UPV [36].

**Figure 9:**
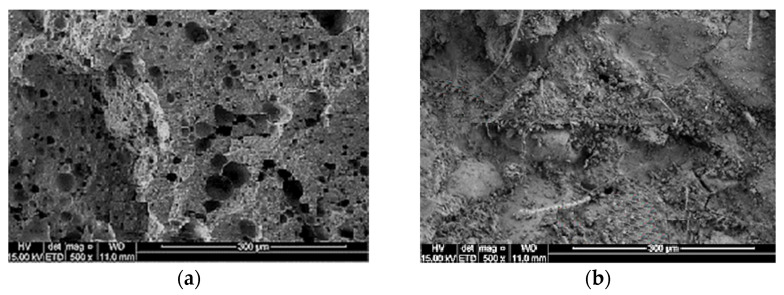
SEM images of LFC. (**a**) control LFC (no fiber inclusion); (**b**) LFC with the inclusion of kenaf fiber.

A solid matrix structure of LFC with the inclusion of fibers in conjunction with separated large air voids, which stem from the added foam, are prerequisites for improved UPV values compared to the control specimen. As illustrated in Figure 9a, it was observed that the control LFC exhibited a porous morphology of varying sizes and had a normal setting between bulk-paste cementitious composites. The high porosity observed can be attributed to initial water absorption, which impacted the rate of hydration. However, LFC with the inclusion of kenaf fiber exhibited smaller voids within the matrix with clearly defined boundaries as shown in Figure 9b. Additionally, microcracks were observed within the control LFC compared with the LFC specimen reinforced with kenaf fiber, where no clear microcracks were detected. This could be attributed to the existence of fibers that alleviated and bridged the creation of the microcracks and prevented them from further propagation [36]. The walls between the air voids comprise the load-carrying frame in the structure of the LFC. It should be pointed out that natural plant fibers are flexible and small in diameter. Essentially, they create a small matrix of aggregates within the larger LFC matrix.

Should an ultrasonic pulse flow via the LFC towards the cementitious paste, a more tortuous route would be generated by the fibers. The more tortuous the path is the longer the traversing time for the ultrasonic pulse. Adding natural fibers allows for the modification of the FC’s pore structure, resulting in decreased gel pores in LFC. Among the four kinds of natural lignocellulosic fiber regarded in this research study, kenaf fiber was found to possess the largest cross-section diameter, i.e., 198 µm, and then ramie (155 µm), jute (121 µm) and hemp fibers (75 µm). Thus, it is clear that the larger the fiber’s diameter is, the result pertaining to the ultrasonic pulse velocity tends to be better.

### 5.3. Water Absorption

**Figure 10:**
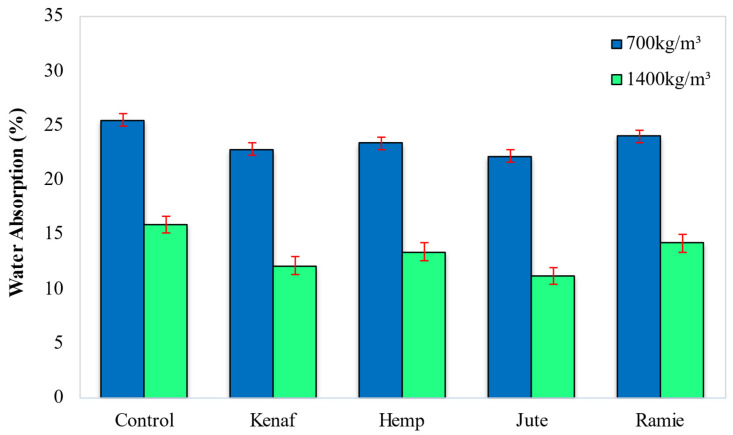
Effect of different types of fiber on LFC water absorption.

Figure 10 demonstrates the water absorption capacity of LFC with the addition of different types of fiber. Figure 10 indicated that the addition of jute fiber led to the optimal results of water absorption at 22.15% and 11.2% for 700 kg/m^3^ and 1400 kg/m^3^ densities, respectively, followed by the percentage of the water absorption with the inclusion of the kenaf fiber at 22.80% and 12.1% for 700 kg/m^3^ and 1400 kg/m^3^ densities, correspondingly. The control mix stemmed from the greatest rate of water absorption of 25.5% and 15.9% for 700 kg/m^3^ and 1400 kg/m^3^ densities respectively. Among all the four fibers, ramie fiber led to the highest water absorption capacity of LFC (24.0%). These trends can be associated with the chemical composition of the fiber. As shown in Table 3, the proportion of cellulose will directly influence the water absorption capacity of fiber-reinforced LFC. For example, the cellulose content in ramie fiber is the highest (70.1%) while cellulose content in jute fiber is the smallest (47.8%). Fiber with high cellulose content has greater transportability hence it attracts more water.

Although, with the inclusion of these natural fibers, the water absorption in LFC can be reduced as compared to the control specimens. Ferreira et al. [36] asserted that natural fiber addition in cement-based material will lead to enhancement in water absorption capacity. This statement is also supported by Kochova et al. [37], that water absorption increases as the fiber content increases. Conversely, moisture diffusion appears by the transmission of fluid fragments over the absorbent structure, even though this process is inhibited by the action of the required number of fibers that fill up the void in the LFC matrix.

However, plentiful fiber can also result in the transport of capillary into the gaps as well as flaws at the interface between the matrix and the fiber. In this process, the impact of water starts with fiber swelling post absorption of moisture and continues along with the matrix micro-cracks that occur around the swollen fibers. Next, the capillary structure, wherein there is a flow of the water molecules along the fiber-matrix edge, results in the dispersion of the water via the bulk matrix. From the fibers, the water-soluble substances leach out, resulting in ultimate fiber-matrix debonding. This could lead to lessening the concrete’s mechanical characteristics.

### 5.4. Porosity

**Figure 11:**
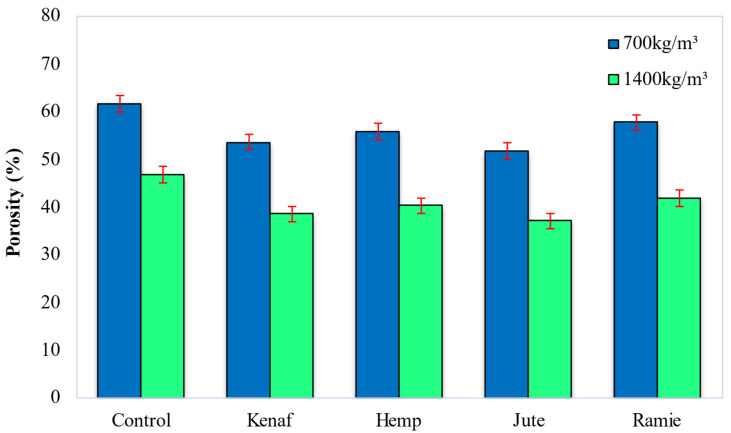
Effect of different types of fiber on porosity of LFC.

Figure 11 shows the effect of different types of fiber on the porosity of LFC. According to Figure 11, the inclusion of all types of natural lignocellulosic fiber resulted in a lower porosity percentage compared to the control specimen. Porosity and void size distribution in LFC may be decreased by using admixtures. Thus, the presence of these natural organic fibers helped initiate blocked pore arrangements to lessen the diameter of openings and diminish the absorptivity of the LFC structure. Hence, it can be assumed that the addition of these fibers plays an important role in reducing the porosity of LFC. Control specimens logged highest porosity of 61.74% and 46.8% for 700 kg/m^3^ and 1400 kg/m^3^ densities, respectively. The inclusion of jute fiber gave the optimal porosity readings of 51.89% and 37.1% for 700 and 1400 kg/m^3^ densities, respectively, while ramie fiber inclusion led to the highest porosity value of 57.76% and 41.9% for 700 kg/m^3^ and 1400 kg/m^3^ densities, correspondingly. The cellulose content in fiber plays a vital role and impacts the porosity of LFC.

Cellulose tends to swell when a process of water absorption is applied to the natural lignocellulosic fibers. Due to the swelling, microcracks could result in a brittle matrix within the cementitious composite pertaining to LFC and could facilitate major transport of water via the fiber matrix boundary and would also yield a high porosity value. Microcracks form in the LFC matrix at the interface region, triggered by cellulose fiber swelling, which could also increase the diffusion transference pertaining to the water through them. In addition, as the capillary mechanism becomes active, the flowing of water molecules via the interface of the fiber-LFC matrix results in higher diffusivity. The difference pertaining to diffusion coefficient values with regards to the same fiber weight fraction in LFC was because of the fiber’s scattering of natural constituents [38].

### 5.5. Porosity-Water Absorption Relationship

**Figure 12:**
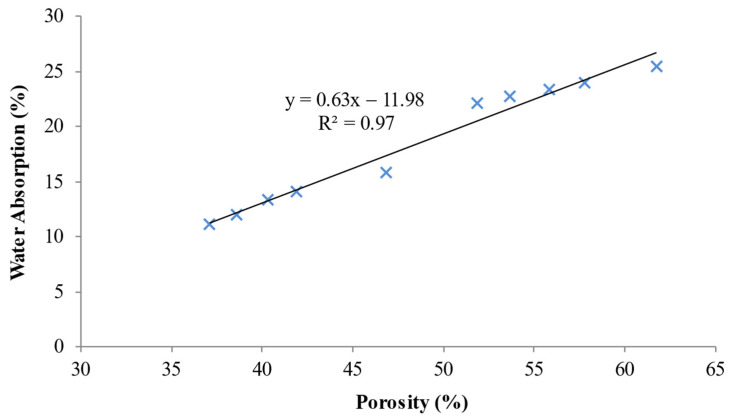
Correlation between the water absorption and porosity of LFC.

Figure 12 displays the connection between porosity and water absorption of LFC. A linear relationship exists between water absorption and the porosity of LFC. The straight-line graph in Figure 12 simply implies that as water absorption increases, the porosity also increases. An R-squared value of 0.97, means that there is a strong linear relationship between water absorption and porosity. The pore structure of the cement matrix modifies both the LFC water absorption capacity and porosity. The dissemination of liquid ensues at the surface and flows into the inner section of the LFC cementitious matrix. This confirms that the surface water absorption has a substantial impact on the porosity of LFC. Furthermore, inner water absorption may have little influence on the LFC porosity.

### 5.6. Porosity-Ultrasonic Pulse Velocity Relationship

**Figure 13:**
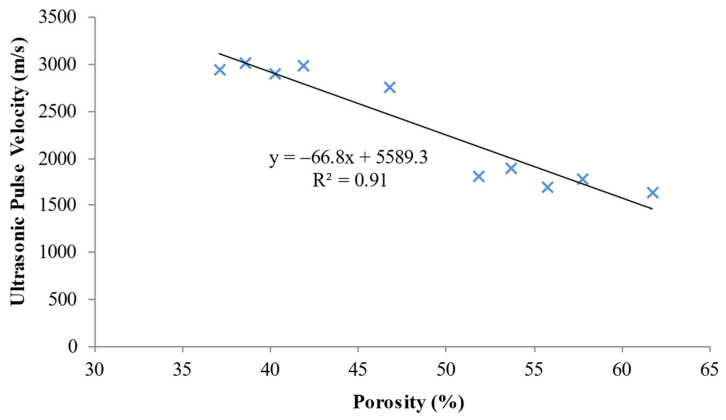
Correlation between the ultrasonic pulse velocity and porosity of LFC.

Figure 13 demonstrates the relationship that exists between LFC’s ultrasonic pulse velocity and porosity. A linear relationship exists between water absorption and the porosity of LFC. The straight-line graph in Figure 13 simply implies that as water absorption increases, the porosity also increases. An R-squared value of 0.97, means that there is a strong linear relationship between water absorption and porosity of LFC. LFC that possesses greater air content (porous) generally possesses more air pores because of the vicinity amongst the air voids, resulting in a higher prevalence pertaining to void merging that leads to the formation of larger irregular air voids. There are a few larger pores available, which could generally be because of the pores mixing as well as in combination with higher levels pertaining to concrete foam. With the addition of natural fibers, there is a reduction in the capillary voids, entrapped air voids, entrained air voids, water voids as well as other heterogeneities, which increase the LFC’s ultrasonic pulse velocity. The wave propagation speed depends on the LFC’s density as well as its flexible characteristic. There is a faster pulse wave routing through a solid medium versus gas and fluid media. In addition, the pulse wave is regarded to be exceptionally complex as well as delicate towards changes in the medium, irrespective of if that occurs via elevating or declining.

## 6. Conclusions

This experimental study aimed to investigate the influence of the addition of natural lignocellulosic fibers on the performance of LFC. LFC mixes comprising kenaf, ramie, hemp and jute fibers of a constant weight fraction of 0.45 were assessed against durability properties comprising flowability, water absorption capability, porosity and ultrasonic pulse velocity (UPV). Two densities of 700 kg/m^3^ and 1400 kg/m^3^ were cast and tested. Based on the analysis performed, the following conclusions can be drawn.

1. Addition of jute fiber led to optimal workability (slump) which recorded a slump of 238 mm and 230 mm for 700 kg/m^3^ and 1400 kg/m^3^ densities, respectively. Concurrently, the lowest slump was recorded for LFC with the addition of ramie fiber (215 mm and 205 mm). The fiber cellulose content had a direct influence on the workability of LFC. Jute fiber had the lowest content of cellulose (47.8%) while the ramie fiber cellulose content was the highest (70.1%). Fiber with a low amount of cellulose content tends to absorb less free water in the mix and put on an ideal slump reading.
2. For ultrasonic pulse velocity (UPV), the best result was accomplished with the presence of kenaf fiber which logged readings of 1897 m/s and 3025 m/s for 700 kg/m^3^ and 1400 kg/m^3^ densities correspondingly. Among the 4 types of fiber considered, the LFC with the inclusion of hemp fiber recorded the lowest UPV reading of 1695 m/s and 2902 m/s for 700 kg/m^3^ and 1400 kg/m^3^ densities respectively. The UPV results can be linked with the fibers’ cross-section diameters. The cross-section diameter of the fibers in the cementitious matrix directly influences the UPV of LFC. For a fiber cross-section of greater diameter, specific cells can fill up the gaps between the void cells of LFC and result in a denser compound, which augmented the LFC’s UPV value.
3. For porosity and water absorption properties, the inclusion of jute fiber led to optimal results while the presence of ramie fiber revealed the highest water absorption and porosity readings, which is unfavorable to LFC. The amount of cellulose in fiber will directly affect the porosity and water absorption of LFC. For instance, among the four fibers employed in this study, the ramie fiber had the highest content of cellulose (70.1%) while cellulose content in jute fiber had the smallest (47.8%). The natural fiber with high cellulose content has greater transportability via the fiber matrix boundary, hence it attracts more water.
4. After all, this preliminary research would support future works on developing more innovative, lighter weight, low-cost, and environment-friendly cement-based materials with the inclusion of natural lignocellulosic fibers in the building and construction sectors.

## Figures and Tables

**Table 1:** The physical properties of OPC and fine sand.

Materials	Density (kg/m^3^)	Specific Surface Area (m^2^/kg)	Specific Gravity
OPC	3.15	300	3.16
Fine Sand	2.11	450	2.61

**Table 2:** Chemical composition of OPC.

Oxide Components	Percentage (%)
Silicon	20.18
Aluminium	4.65
Ferrous	3.59
Calcium	62.59
Magnesium	1.56
Sulphur	2.67
Sodium	0.23
Potassium	0.74
Chloride	0.03
Free calcium	1.21
Loss of ignition	1.23
Insoluble residue	1.32

**Table 3:** Chemical composition of natural lignocellulosic fibers.

Composition	Kenaf	Hemp	Jute	Ramie
Cellulose (%)	63.5	65.1	47.8	70.1
Hemicellulose (%)	14.2	8.2	23.5	10.2
Lignin (%)	11.3	14.7	17.8	9.3
Ash (%)	4.7	2.6	0.0	2.1
Pectin (%)	0.0	0.8	0.3	1.1
Wax (%)	0.1	0.6	0.5	0.3
Moisture (%)	6.2	8.0	10.1	6.9

**Table 4:** Physical properties of natural lignocellulosic fibers.

Physical Properties	Kenaf	Hemp	Jute	Ramie
Diameter (µm)	198	75	121	155
Fiber length (mm)	25	25	25	25
Density (kg/m^3^)	1550	920	1380	1470
Micro-fibrillar angle (°)	7.0	3.5	8.0	7.5

**Table 5:** Mechanical properties of natural lignocellulosic fibers.

Mechanical Properties	Kenaf	Hemp	Jute	Ramie
Tensile strength (MPa)	297.8	192.4	121.8	321.5
Young’s modulus (GPa)	19.8	14.5	12.23	16.7
Torsional Rigidity (10^−9^ N/m^2^)	1.56	1.19	0.82	1.35
Elongation at break (%)	6.56	9.96	11.45	8.95
Poisson’s ratio (v)	0.40	0.38	0.35	0.45

**Table 6:** Mix proportions of LFC.

Dry Density (kg/m^3^)	Fiber Types	Mix Ratio (C:S:W)	Fiber (kg)	Cement (kg)	Fine Sand (kg)	Water (kg)
700	-	1:1.5:0.45	0.00	26.63	39.95	11.99
700	Jute	1:1.5:0.45	0.37	26.63	39.95	11.99
700	Kenaf	1:1.5:0.45	0.37	26.63	39.95	11.99
700	Hemp	1:1.5:0.45	0.37	26.63	39.95	11.99
700	Ramie	1:1.5:0.45	0.37	26.63	39.95	11.99
1400	-	1:1.5:0.45	0.00	51.91	77.87	23.36
1400	Jute	1:1.5:0.45	0.68	51.91	77.87	23.36
1400	Kenaf	1:1.5:0.45	0.68	51.91	77.87	23.36
1400	Hemp	1:1.5:0.45	0.68	51.91	77.87	23.36
1400	Ramie	1:1.5:0.45	0.68	51.91	77.87	23.36

## Data Availability

Not applicable.
